# Performance Comparison of Relay-Based Covert Communications: DF, CF and AF

**DOI:** 10.3390/s23218747

**Published:** 2023-10-26

**Authors:** Jihwan Moon

**Affiliations:** Department of Mobile Convergence Engineering, Hanbat National University, Daejeon 34158, Republic of Korea; anschino@staff.hanbat.ac.kr

**Keywords:** physical layer security, covert communications, low probability of detection, decode-and-forward, compress-and-forward, amplify-and-forward, relay

## Abstract

In this paper, we investigate the performance of covert communications in different types of a relay system: decode-and-forward (DF), compress-and-forward (CF) and amplify-and-forward (AF). We consider a source node that attempts to send both public and covert messages to a destination node through a relay on which a covert message detector is embedded. By taking the minimum detection error probability (DEP) at the relay into account, we optimize the power distribution between the public and covert messages to achieve the maximum covert rate. We further make a delay-aware comparison among DF, CF and AF relay systems with the obtained closed-form covert rates and conduct an extensive examination on the asymptotic behaviors in different limits. Our analyses reveal that CF or AF tend to outperform DF for high source transmit power or low relay transmit power, while various system parameters such as the processing delay, minimum required quality of service for public messages and DEP threshold lead to different performance relationships among DF, CF and AF for high relay transmit power. Numerical results verify our investigation into the performance comparison in various channel models.

## 1. Introduction

Recent advancements in wireless technology have opened up possibilities to enhance our daily lives. These include applications such as vehicle-to-everything (V2X) communication, low Earth orbit (LEO) satellites, dynamic public safety networks, and the Internet of Things (IoT) [1,2]. Meanwhile, as wireless communications technology thrives, the apprehension about safeguarding confidential data is steadily on the rise [3]. Cryptographic methods have traditionally been deemed highly efficient in terms of defense [4], but they come with certain drawbacks, such as the intricate process of generating secret keys and susceptibility to eavesdroppers equipped with superior computational capabilities. This has led to the emergence of physical layer security as an alternative approach [5]. Its primary advantage lies in its ability to thwart eavesdroppers effectively in wireless connections between legitimate parties and unauthorized entities. This is achieved through techniques including nullifying beamforming using multiple antennas or introducing artificial noise, which potentially mitigates the weaknesses associated with cryptography [6].

While the integration of cryptography and physical layer security can effectively prevent eavesdropping, there is a need for an even higher level of security where the existence of communications should remain hidden [7]. Particularly, even if the content of information is unavailable, adversaries may still conduct traffic analysis to gather metadata such as source of packets, the frequency at which request and response packets are transmitted, and even visible e-mail addresses [4]. These challenges have given rise to the concept of *covert communications* or *low-probability-of-detection* communications [7,8].

The authors in [8] investigated a primitive form of covert communications which consists of a covert transmitter, receiver and a warden. They claimed that a positive covert rate is achieved if the transmitter concurrently transmits public and covert messages, when there exists uncertainty in channel state information (CSI) or in noise level.

The applicability of covert communications has been widely studied in various relay-based communications systems as well. In [9], the authors presented two covert transmission strategies for a greedy amplify-and-forward (AF) half-duplex (HD) relay, which opportunistically transmits a covert message alongside the public message. In a similar context, the authors of [10,11] explored a multi-antenna decode-and-forward (DF) relay and a self-sustained AF relay using time switching and power splitting for energy harvesting, respectively. The work in [12] introduced an AF full-duplex (FD) relay, while [13] presented an AF joint FD/HD relay to support covert transmission. Additionally, [14] examined a two-way AF greedy relay that opportunistically sends covert messages, and [15] explored two-way intelligent reflecting surface-based covert communications. For multi-antenna DF relay-assisted covert communications, [16] analyzed achievable covert rates, considering both direct and relay links. Furthermore, secure communications with covertness requirements were addressed in [17] in the presence of an untrusted relay, and [18] discussed a similar system with an external eavesdropper, incorporating practical assumptions such as warden location uncertainty.

A majority of relay-based covert communications assume that a covert message originates either from relays and is sent on top of the source node [9,10,11,14,19,20] or from the source node such that the whole end-to-end communication needs to be hidden from wardens [12,13,15,16,21,22]. On the other hand, it is also possible that the source node transmits both public and covert messages in a way that only the covert portion is kept undetected [23]. Such a strategy is necessary when an entity is surrounded by adversaries and wishes to carry out a covert mission in disguise. However, there are a limited number of works on this type of a covert transmission, which requires more attention from the field.

In the fifth-generation (5G) and future communication networks, it is also worth noting that there is an opportunity to create an architecture that utilizes cloud processing for collaborative interference control and centralized computation. Depending on the hardware specifications and service requirements, the process of delivering content from the cloud to the user may employ rapid relaying protocols such as AF and compress-and-forward (CF) or a time-consuming but possibly more stable method such as DF. Consequently, it is essential to conduct a comprehensive examination of the potential for covert communications in DF, CF and AF relay systems. Our previous work [24] studied for the first time a CF relay-based covert communications system and a condition in which the CF and AF schemes become equivalent at optimum. Still, to the best of our knowledge, there are not sufficient studies on a performance comparison among DF, CF and AF relay-based covert communications.

In this paper, we generalize the system model of [24] to encompass DF, CF and AF relay protocols. To be specific, a source node that attempts to send both public and covert messages to a destination node through either a DF, CF or AF relay on which a covert message detector is embedded. By taking the minimum detection error probability (DEP) at the relay into account, we obtain the optimal power distribution between the public and covert messages to achieve the maximum covert rate for each relay type. We further make a delay-aware comparison among DF, CF and AF relay systems with closed-form covert rates and conduct an comprehensive examination on the asymptotic behaviors in different limits. Our analyses show that CF or AF tend to outperform DF for high source transmit power, while various system parameters such as the processing delay, minimum required quality of service for public messages and DEP threshold lead to different performance relationships among DF, CF and AF for high relay transmit power. Numerical results verify our investigation on the performance comparison. Our contributions can be summarized as follows:

- Besides the CF and AF relays explored in [24], we provide in this paper the optimal public and covert rates of DF relay-based covert communications for completeness. We optimize the power distribution between the public and covert messages and obtain a closed-form expression of the achievable covert rate.
- Noting that DF, CF and AF relays undergo different processing delays in practice, we develop upon the results of [24] and this paper delay-aware expressions of the achievable covert rate for each type of relays by adopting the delay relationship in [25].
- We then examine and make a delay-aware comparison among the asymptotic behaviors of the achievable covert rates with DF, CF and AF relays in different limits of source and relay transmit power for practical usefulness.
- Our analyses reveal that CF or AF tend to outperform DF for high source transmit power or low relay transmit power, while various system parameters such as the processing delay, minimum required quality of service for public messages and DEP threshold lead to different performance relationships among DF, CF and AF for high relay transmit power.
- We conduct various numerical examples, and they are in congruence with our studies on the asymptotic behaviors in various channel models.
- The results of this paper can provide a useful guideline in an environment where multiple relays with different forwarding protocols exist or where a single relay is capable of selecting either DF, CF or AF. We suggest such covert communications scenarios as interesting future works.

## 2. System Model

### 2.1. Received Signals

**Figure 1:**
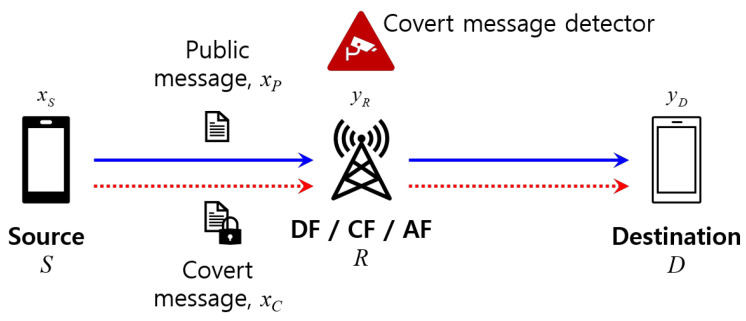
System model.

Figure 1 illustrates the system model under consideration, where the source node *S* and the destination node *D* communicates via the relay *R*. We make an assumption that a direct link between the source and destination nodes is absent due to environmental conditions, such as being located in shadowed areas or being separated by a considerable distance. In addition to transmitting public messages, the source node also attempts to transmit a covert message and wishes that the covert message detector incorporated within the relay fails to identify it.

The received signal at the relay is written as

(1) $$\begin{array}{c} y_{R}=h_{SR}\sqrt{P_{S}}\left(\sqrt{\alpha }x_{P}+\sqrt{1-\alpha }x_{C}\right)+z_{R}, \end{array}$$

where $x_{P}∼\mathrm{CN}\left(0,1\right)$ and $x_{C}∼\mathrm{CN}\left(0,1\right)$ indicate the public and covert messages, respectively, $P_{S}$ means the source transmit power, α controls the proportion of $P_{S}$ for $x_{P}$, and $z_{R}∼\mathrm{CN}\left(0,\sigma_{R}^{2}\right)$ denotes the additive noise. As in [26,27], we assume that the noise $\sigma_{R}^{2}$ varies uncertainly in this work such that $\sigma_{R,\mathrm{dB}}^{2}∼U\left({\bar{\sigma }}_{R,\mathrm{dB}}^{2}-\zeta_{\mathrm{dB}},{\bar{\sigma }}_{R,\mathrm{dB}}^{2}+\zeta_{\mathrm{dB}}\right)$ in decibel range. Here, ${\bar{\sigma }}_{R,\mathrm{dB}}^{2}$ and $\zeta_{\mathrm{dB}}\geq 0$ stand for the mean and bounded range, respectively. It is assumed that all nodes have access to the global CSI since covert communications are carried out under the normal operation of relay.

We now consider three different types of relays, DF, CF and AF, in the following and derive the expressions for both the public and covert rates at the destination node.

#### 2.1.1. DF Relay

We first note from (1) that the achievable rate for the combined message $x_{S}≜\sqrt{\alpha }x_{P}+\sqrt{1-\alpha }x_{C}$ in the *S*-*R* hop is written by

(2) $$\begin{array}{c} {\bar{r}}_{S}=\log_{2}\left(1+\frac{{\left|h_{SR}\right|}^{2}P_{S}}{\sigma_{R}^{2}}\right). \end{array}$$

Once the DF relay succeeds in decoding $x_{S}$, it is forwarded to the destination node, and the received signal can be shown as

(3) $$\begin{array}{c} y_{D}=h_{RD}\sqrt{P_{R}}\left(\sqrt{\alpha }x_{P}+\sqrt{1-\alpha }x_{C}\right)+z_{D}, \end{array}$$

where $P_{R}$ is the relay transmit power. The resulting achievable rate for $x_{S}$ in the *R*-*D* hop is accordingly given by

(4) $$\begin{array}{c} {\bar{r}}_{R}=\log_{2}\left(1+\frac{{\left|h_{RD}\right|}^{2}P_{R}}{\sigma_{D}^{2}}\right). \end{array}$$

It is evident from (2) and (4) that the actual data rate for $x_{S}$ is upper bounded by ${\bar{r}}_{S}$ and ${\bar{r}}_{R}$ for successful decoding at the relay and destination node.

The destination node first decodes the public message by taking the covert message as interference [28], leading to the achievable rate for the public message of

(5) $$\begin{array}{c} r_{P,\mathrm{DF}}=\log_{2}\left(1+\frac{{\left|h_{RD}\right|}^{2}P_{R}\alpha }{{\left|h_{RD}\right|}^{2}P_{R}\left(1-\alpha \right)+\sigma_{D}^{2}}\right). \end{array}$$

Subsequently, the destination node recovers the covert message by removing the decoded public message, and the achievable rate for the covert message is derived as

(6) $$\begin{array}{c} r_{C,\mathrm{DF}}=\log_{2}\left(1+\frac{{\left|h_{RD}\right|}^{2}P_{R}\left(1-\alpha \right)}{\sigma_{D}^{2}}\right). \end{array}$$

It is also clear that the actual data rates for $x_{P}$ and $x_{C}$ are limited by $r_{P,\mathrm{DF}}$ and $r_{C,\mathrm{DF}}$, respectively.

#### 2.1.2. CF Relay

In the presence of the finite wireless link capacity ${\bar{r}}_{R}$ between the CF relay and destination node, the CF relay compresses the signal $y_{R}$ to a lower-resolution form ${\tilde{y}}_{R}$. Previous works in [29,30] have demonstrated that the Gaussian model of the quantization noise ensures the existence of a quantization codebook as long as the mutual information $I\left(y_{R};{\tilde{y}}_{R}\right)$ is less than or equal to the data rate. Consequently, we define $q_{R}≜y_{R}-{\tilde{y}}_{R}≃CN\left(0,Q_{R}\right)$, where $Q_{R}$ signifies the degree of compression. The successful decompression condition at the destination node is then described by $I\left(y_{R};{\tilde{y}}_{R}\right)\leq {\bar{r}}_{R}$ where

(7) $$\begin{array}{cc} \; & I\left(y_{R};{\tilde{y}}_{R}\right)=\log_{2}\left(1+\frac{{\left|h_{SR}\right|}^{2}P_{S}+\sigma_{R}^{2}}{Q_{R}}\right). \end{array}$$

After ${\tilde{y}}_{R}$ is successfully decompressed, the destination node decodes the public and covert messages successively as described in Section 2.1.1. Thus, the achievable rates for the public and covert messages can be calculated in a similar manner by

(8) $$\begin{array}{cc} \; & r_{P,\mathrm{CF}}=\log_{2}\left(1+\frac{{\left|h_{SR}\right|}^{2}P_{S}\alpha }{{\left|h_{SR}\right|}^{2}P_{S}\left(1-\alpha \right)+\sigma_{R}^{2}+Q_{R}}\right), \end{array}$$

(9) $$\begin{array}{cc} \; & r_{C,\mathrm{CF}}=\log_{2}\left(1+\frac{{\left|h_{SR}\right|}^{2}P_{S}\left(1-\alpha \right)}{\sigma_{R}^{2}+Q_{R}}\right). \end{array}$$

#### 2.1.3. AF Relay

The destination node receives an amplified version of $y_{R}$ from the AF relay as

(10) $$\begin{array}{c} y_{D}=h_{RD}\sqrt{P_{R}}x_{R}+z_{D}, \end{array}$$

where $x_{R}≜y_{R}/\sqrt{{\left|h_{SR}\right|}^{2}P_{S}+\sigma_{R}^{2}}$ represents the normalized unit-power signal from the AF relay. With some manipulations, we can derive the similar achievable rates for the public and covert messages to (5) or (8), and (6) or (9) as

(11) $$\begin{array}{cc} \; & r_{P,\mathrm{AF}}=\log_{2}\left(1+\frac{{\left|h_{SR}\right|}^{2}P_{S}\alpha }{{\left|h_{SR}\right|}^{2}P_{S}\left(1-\alpha \right)+\sigma_{R}^{2}+{\tilde{\sigma }}_{D}^{2}}\right), \end{array}$$

(12) $$\begin{array}{cc} \; & r_{C,\mathrm{AF}}=\log_{2}\left(1+\frac{{\left|h_{SR}\right|}^{2}P_{S}\left(1-\alpha \right)}{\sigma_{R}^{2}+{\tilde{\sigma }}_{D}^{2}}\right), \end{array}$$

respectively, where ${\tilde{\sigma }}_{D}^{2}≜\left({\left|h_{SR}\right|}^{2}P_{S}+\sigma_{R}^{2}\right)\sigma_{D}^{2}/\left({\left|h_{RD}\right|}^{2}P_{R}\right)$.

### 2.2. Covert Message Detection

The covert message detector at the relay is designed to identify the presence of any additional messages apart from the public message. To achieve this, it first removes the public message from the received signal $y_{R}$. This process results in an effective residual signal ${\tilde{z}}_{R}≜y_{R}-h_{SR}\sqrt{P_{S}}x_{P}$ assuming that the relay perfectly knows $h_{SR}$ and $P_{S}$ [31]. We then establish null and alternative hypotheses as

(13) $$\begin{array}{c} \begin{array}{cc} H_{0}\;\;:\;\; & \;\;{\tilde{z}}_{R}\;=\;z_{R},\\ H_{1}\;\;:\;\; & \;\;{\tilde{z}}_{R}\;=\;h_{SR}\sqrt{P_{S}}\left(\left(\sqrt{\alpha }-1\right)x_{P}+\sqrt{1-\alpha }x_{C}\right)+z_{R}, \end{array} \end{array}$$

where the null hypothesis $H_{0}$ represents an event that the source node did not transmit a covert message, and the alternative hypothesis $H_{1}$ denotes an event that a covert message exists. With a radiometer [26] as a detection measure, the detector can utilize the sufficient test statistic *T* for (13) after collecting an N→∞ number of ample signals, which reduces to the average power $E[{\left|{\tilde{z}}_{R}\right|}^{2}]$ as

(14) $$\begin{array}{c} \begin{array}{cc} H_{0}: & T=\sigma_{R}^{2},\\ H_{1}: & T=2{\left|h_{SR}\right|}^{2}P_{S}\left(1-\sqrt{\alpha }\right)+\sigma_{R}^{2}, \end{array} \end{array}$$

and the covert message detector decides that a covert link exists if T≥τ for some threshold τ.

The DEP $\mathrm{Pr}\left(e\right)$ is composed of false alarm and miss probabilities as

(15) $$\begin{array}{c} \mathrm{Pr}\left(e\right)\;\;=\;\;\underset{\mathrm{False}\;\mathrm{alarm}}{\underset{︸}{\mathrm{Pr}\left(T\;\geq \;\tau |H_{0}\right)}}\mathrm{Pr}\left(H_{0}\right)\;+\;\underset{\mathrm{Miss}}{\underset{︸}{\mathrm{Pr}\left(T\;<\;\tau |H_{1}\right)}}\mathrm{Pr}\left(H_{1}\right), \end{array}$$

where the detector assumes that the covert transmission occurs at random, i.e., $\mathrm{Pr}\left(H_{0}\right)=\mathrm{Pr}\left(H_{1}\right)=0.5$. The optimal τ minimizing the DEP can be obtained from [24] as

(16) $$\begin{array}{c} \tau^{★}=2{\left|h_{SR}\right|}^{2}P_{S}\left(1-\sqrt{\alpha }\right)+\frac{1}{\zeta }{\bar{\sigma }}_{R}^{2}, \end{array}$$

and the corresponding minimum DEP is also given by [24]

(17) $$\begin{array}{c} {\left.\mathrm{Pr}\left(e\right)\right|}_{\tau =\tau^{★}}=\frac{1}{2}\left(1-\frac{1}{2\ln\zeta }\left(\ln\frac{\tau^{★}}{\tau^{★}-2{\left|h_{SR}\right|}^{2}P_{S}\left(1-\sqrt{\alpha }\right)}\right)\right), \end{array}$$

as long as $\zeta {\bar{\sigma }}_{R}^{2}\geq 2{\left|h_{SR}\right|}^{2}P_{S}\left(1-\sqrt{\alpha }\right)+{\bar{\sigma }}_{R}^{2}/\zeta$. Note that (16) yields the worst-case minimum DEP assuming that the detector uses the exact value of α.

## 3. Problem Formulation

To make a performance comparison among the three types of relay schemes, we first need to identify the optimal power distribution between the public and covert messages that maximizes the covert rate. Depending on the type of relay, we formulate optimization problems as discussed below.

### 3.1. DF Relay

First, when the DF relay is considered, we solve

(18a) $$\begin{array}{cc} (P1):\; & \max_{\alpha ,b_{P},b_{C}}\;b_{C}, \end{array}$$

(18b) $$\begin{array}{cc} \mathrm{subject}\;\mathrm{to}:\; & b_{P}\geq {\bar{r}}_{P}, \end{array}$$

(18c) $$\begin{array}{cc} & b_{P}+b_{C}\leq \min\left({\left.{\bar{r}}_{S}\right|}_{\sigma_{R}^{2}=\zeta {\bar{\sigma }}_{R}^{2}},{\bar{r}}_{R}\right), \end{array}$$

(18d) $$\begin{array}{cc} \; & b_{P}\leq r_{P,\mathrm{DF}}, \end{array}$$

(18e) $$\begin{array}{cc} \; & b_{C}\leq r_{C,\mathrm{DF}}, \end{array}$$

(18f) $$\begin{array}{cc} \; & {\left.\mathrm{Pr}\left(e\right)\right|}_{\tau =\tau^{★}}\geq \varepsilon , \end{array}$$

(18g) $$\begin{array}{cc} \; & \zeta {\bar{\sigma }}_{R}^{2}\geq 2{\left|h_{SR}\right|}^{2}P_{S}\left(1-\sqrt{\alpha }\right)+\frac{1}{\zeta }{\bar{\sigma }}_{R}^{2}, \end{array}$$

(18h) $$\begin{array}{cc} \; & 0\leq \alpha \leq 1, \end{array}$$

where $b_{P}$ and $b_{C}$ denote the actual rates for public and covert messages, respectively. We impose the minimum quality of service ${\bar{r}}_{P}$ on $b_{P}$ in (18b) and the upper bounds discussed in Section 2.1.1 on both of $b_{P}$ and $b_{C}$ in (18c)–(18e). We also emphasize that (P1) provides the worst-case performance of the covert rate by considering the conservative constraint in (18c). To be specific, we take the lowest possible ${\bar{r}}_{S}$ into account by setting $\sigma_{R}^{2}=\zeta {\bar{\sigma }}_{R}^{2}$. Constraints (18f) and (18g) guarantee a positive minimum DEP for 0≤ε≤0.5, and (18h) sets a feasible region for α.

### 3.2. CF Relay

When the CF relay is implemented, we tackle

(19a) $$\begin{array}{cc} (P2):\; & \max_{\alpha ,Q_{R}}\;{\left.r_{C,\mathrm{CF}}\right|}_{\sigma_{R}^{2}=\zeta {\bar{\sigma }}_{R}^{2}}, \end{array}$$

(19b) $$\begin{array}{cc} \mathrm{subject}\;\mathrm{to}:\; & {\left.I\left(y_{R};{\tilde{y}}_{R}\right)\right|}_{\sigma_{R}^{2}=\zeta {\bar{\sigma }}_{R}^{2}}\leq {\bar{r}}_{R}, \end{array}$$

(19c) $$\begin{array}{cc} \; & {\left.r_{P,\mathrm{CF}}\right|}_{\sigma_{R}^{2}=\zeta {\bar{\sigma }}_{R}^{2}}\geq {\bar{r}}_{P}, \end{array}$$

(19d) $$\begin{array}{cc} \; & {\left.\mathrm{Pr}\left(e\right)\right|}_{\tau =\tau^{★}}\geq \varepsilon , \end{array}$$

(19e) $$\begin{array}{cc} \; & \zeta {\bar{\sigma }}_{R}^{2}\geq 2{\left|h_{SR}\right|}^{2}P_{S}\left(1-\sqrt{\alpha }\right)+\frac{1}{\zeta }{\bar{\sigma }}_{R}^{2}, \end{array}$$

(19f) $$\begin{array}{cc} \; & 0\leq \alpha \leq 1,\;Q_{R}\geq 0. \end{array}$$

(P2) also provides the worst-case performance by maximizing the worst-case covert rate in (19a) and taking the worst-case compression into consideration such that the minimum amount of quantization error becomes the largest in (19b), both by setting the noise variance at the CF relay to $\sigma_{R}^{2}=\zeta {\bar{\sigma }}_{R}^{2}$. The minimum guaranteed public rate threshold ${\bar{r}}_{P}$ is imposed on (19c), and Constraints (19d) and (19e) assure a positive minimum DEP for 0≤ε≤0.5. Lastly, (19f) indicates general feasible regions for α and $Q_{R}$.

### 3.3. AF Relay

For the AF relay, we optimize

(20a) $$\begin{array}{cc} (P3):\; & \max_{\alpha }\;{\left.r_{C,\mathrm{AF}}\right|}_{\sigma_{R}^{2}=\zeta {\bar{\sigma }}_{R}^{2}}, \end{array}$$

(20b) $$\begin{array}{cc} \mathrm{subject}\;\mathrm{to}:\; & {\left.r_{P,\mathrm{AF}}\right|}_{\sigma_{R}^{2}=\zeta {\bar{\sigma }}_{R}^{2}}\geq {\bar{r}}_{P}, \end{array}$$

(20c) $$\begin{array}{cc} \; & {\left.\mathrm{Pr}\left(e\right)\right|}_{\tau =\tau^{★}}\geq \varepsilon , \end{array}$$

(20d) $$\begin{array}{cc} \; & \zeta {\bar{\sigma }}_{R}^{2}\geq 2{\left|h_{SR}\right|}^{2}P_{S}\left(1-\sqrt{\alpha }\right)+\frac{1}{\zeta }{\bar{\sigma }}_{R}^{2}, \end{array}$$

(20e) $$\begin{array}{cc} \; & 0\leq \alpha \leq 1. \end{array}$$

Similar to (P2), we maximize the worst-case covert rate in (20a) subject to the worst-case rate for public messages in (20b) by setting the noise variance at the AF relay to $\sigma_{R}^{2}=\zeta {\bar{\sigma }}_{R}^{2}$. Constraints (20c)–(20e) are equivalent to (P1) and (P2).

## 4. Proposed Solutions

In this section, we provide the optimal solutions for (P1), (P2) and (P3).

### 4.1. DF Relay

(P1) reveals that the minimum quality of service for public messages ${\bar{r}}_{P}$ should satisfy ${\bar{r}}_{P}\leq \min\left({\bar{r}}_{S}{|}_{\sigma_{R}^{2}=\zeta {\bar{\sigma }}_{R}^{2}},{\bar{r}}_{R}\right)$ to ensure the feasibility, which we assume throughout this subsection.

Constraints (18f) and (18g) can be rewritten by

(21) $$\begin{array}{cc} \; & \sqrt{\alpha }\geq 1-\left(\zeta^{1-4\varepsilon }-\frac{1}{\zeta }\right)\frac{{\bar{\sigma }}_{R}^{2}}{2{\left|h_{SR}\right|}^{2}P_{S}}, \end{array}$$

(22) $$\begin{array}{cc} \; & \sqrt{\alpha }\geq 1-\left(\zeta -\frac{1}{\zeta }\right)\frac{{\bar{\sigma }}_{R}^{2}}{2{\left|h_{SR}\right|}^{2}P_{S}}, \end{array}$$

respectively. It is clear that (22) is automatically fulfilled when (21) is satisfied for 0≤ε≤0.5. Hence, Constraints (18f)–(18h) are reduced to $\bar{\alpha }\leq \alpha \leq 1$ with

(23) $$\begin{array}{c} \bar{\alpha }≜{\left(\max\left(1-\left(\zeta^{1-4\varepsilon }-\frac{1}{\zeta }\right)\frac{{\bar{\sigma }}_{R}^{2}}{2{\left|h_{SR}\right|}^{2}P_{S}},0\right)\right)}^{2}. \end{array}$$

We also note that decreasing $b_{P}$ enlarges the feasible region of $b_{C}$ in (18c) and α in (18d). Therefore, we may simply set the optimal $b_{P}$ to the minimum required rate from (18b), i.e.,

(24) $$\begin{array}{c} b_{P}^{★}={\bar{r}}_{P}, \end{array}$$

without loss of optimality.

With these in hand, (P1) is reformulated into

(25a) $$\begin{array}{cc} (P1.1):\; & \max_{\alpha ,b_{C}}\;b_{C}, \end{array}$$

(25b) $$\begin{array}{cc} \mathrm{subject}\;\mathrm{to}:\; & b_{C}\leq \min\left({\left.{\bar{r}}_{S}\right|}_{\sigma_{R}^{2}=\zeta {\bar{\sigma }}_{R}^{2}}-{\bar{r}}_{P},{\bar{r}}_{R}-{\bar{r}}_{P},r_{C,\mathrm{DF}}\right), \end{array}$$

(25c) $$\begin{array}{cc} \; & \alpha \geq \frac{\left(2^{{\bar{r}}_{P}}-1\right)\left({\left|h_{RD}\right|}^{2}P_{R}+\sigma_{D}^{2}\right)}{2^{{\bar{r}}_{P}}{\left|h_{RD}\right|}^{2}P_{R}}, \end{array}$$

(25d) $$\begin{array}{cc} \; & \bar{\alpha }\leq \alpha \leq 1, \end{array}$$

where Constraint (25b) comes from merging (18c) and (18e), and (25c) is a re-expression of (18d), both using (24). Noticing that the upper bound of $b_{C}$ on the right hand side in (25b) is a decreasing function of α from (6), we can conclude that the optimal α should be as low as possible. That is,

(26) $$\begin{array}{c} \alpha_{\mathrm{DF}}^{★}=\min\left(\max\left(\frac{\left(2^{{\bar{r}}_{P}}-1\right)\left({\left|h_{RD}\right|}^{2}P_{R}+\sigma_{D}^{2}\right)}{2^{{\bar{r}}_{P}}{\left|h_{RD}\right|}^{2}P_{R}},\bar{\alpha }\right),1\right), \end{array}$$

and consequently,

(27) $$\begin{array}{cc} b_{C}^{★} & =\min\left({\left.{\bar{r}}_{S}\right|}_{\sigma_{R}^{2}=\zeta {\bar{\sigma }}_{R}^{2}}-{\bar{r}}_{P},{\bar{r}}_{R}-{\bar{r}}_{P},{\left.r_{C,\mathrm{DF}}\right|}_{\alpha =\alpha^{★}}\right),\\ \; & =\min\left(\log_{2}\left(1+\gamma_{SR,\min}\right)-{\bar{r}}_{P},\log_{2}\left(1+\gamma_{RD}\right)-{\bar{r}}_{P},\log_{2}\left(1+\gamma_{RD}\left(1-\bar{\alpha }\right)\right)\right), \end{array}$$

where $\gamma_{SR,\min}≜{\left|h_{SR}\right|}^{2}P_{S}/\left(\zeta {\bar{\sigma }}_{R}^{2}\right)$ and $\gamma_{RD}≜{\left|h_{RD}\right|}^{2}P_{R}/\left(\sigma_{D}^{2}\right)$. It is worth noting that the optimal achievable covert rate is, accordingly, $r_{C,\mathrm{DF}}^{★}=b_{C}^{★}$.

### 4.2. CF Relay

From our previous work [24], the optimal solutions for (P2) are obtained as

(28) $$\begin{array}{cc} \; & Q_{R}^{★}=\frac{\left({\left|h_{SR}\right|}^{2}P_{S}+\zeta {\bar{\sigma }}_{R}^{2}\right)\sigma_{D}^{2}}{{\left|h_{RD}\right|}^{2}P_{R}}, \end{array}$$

(29) $$\begin{array}{cc} \; & \alpha_{\mathrm{CF}}^{★}\;\;=\;\;\min\;\left(\;\max\;\left(\;\frac{\left({\left|h_{SR}\right|}^{2}P_{S}\;+\;\zeta {\bar{\sigma }}_{R}^{2}\;+\;Q_{R}^{★}\right)\left(2^{{\bar{r}}_{P}}\;-\;1\right)}{{\left|h_{SR}\right|}^{2}P_{S}2^{{\bar{r}}_{P}}},\bar{\alpha }\;\right)\;,1\;\right)\;, \end{array}$$

which yields

(30) $$\begin{array}{c} r_{C,\mathrm{CF}}^{★}=\min\left(\log_{2}\left(1+\frac{\gamma_{SR,\min}}{1+\left(\gamma_{SR,\min}+1\right)\gamma_{RD}^{-1}}\right)-{\bar{r}}_{P},\log_{2}\left(1+\frac{\gamma_{SR,\min}\left(1-\bar{\alpha }\right)}{1+\left(\gamma_{SR,\min}+1\right)\gamma_{RD}^{-1}}\right)\right). \end{array}$$

We refer readers to [24] for detail.

### 4.3. AF Relay

In our previous work [24], we discovered that the optimal power allocation and the resultant covert rates for our considered CF and AF relay systems coincide with each other, i.e.,

(31) $$\begin{array}{c} \alpha_{AF}^{★}=\alpha_{CF}^{★}, \end{array}$$

for (P3). It is easily seen that the CF public rate $r_{P,\mathrm{CF}}$ in (8) and covert rate $r_{C,\mathrm{CF}}$ in (9) are equivalent to (11) and (12), respectively, when the CF relay employs an adequate quantization strategy with the optimal quantization noise $Q_{R}^{★}$ in (28), i.e., $Q_{R}^{★}={\tilde{\sigma }}_{D}^{2}$. The resulting optimal covert rate for the AF relay is accordingly given by

(32) $$\begin{array}{c} r_{C,\mathrm{AF}}^{★}=r_{C,\mathrm{CF}}^{★}. \end{array}$$

We refer readers to [24] for detail.

## 5. Performance Comparison with Relay Processing Delay

We now examine the optimal covert rates of different types of relays by taking the processing delay into account. Since Section 4.3 and [24] revealed that the optimal power distributions and covert rates are equivalent for AF and CF relays, here, we concentrate on the comparison between DF and AF assuming that the delay difference the AF and CF is negligible.

The authors in [25] developed the relationship between the codeword lengths of DF relay $L_{DF}$ and AF relay $L_{AF}$ that yield the same processing delay by

(33) $$\begin{array}{c} L_{AF}≃\frac{2\left(1+\delta \right)}{2+\delta }L_{DF} \end{array}$$

in the moderate to high transmit power regime for a delay factor δ≥0. We note that δ=0 indicates an equal processing delay while high δ means a larger difference in processing time between DF and AF. Utilizing (33), the public and covert rates between DF and AF are related by

(34) $$\begin{array}{c} r_{P,\mathrm{DF}}=\frac{2+\delta }{2\left(1+\delta \right)}r_{P,\mathrm{AF}}\;\mathrm{and}\;r_{C,\mathrm{DF}}=\frac{2+\delta }{2\left(1+\delta \right)}r_{C,\mathrm{AF}}. \end{array}$$

We can thus conduct a delay-aware comparison if ${\bar{r}}_{P}$ of (P1) is first replaced by ${\bar{r}}_{P}\left(2\left(1+\delta \right)\right)/\left(2+\delta \right)$, and the obtained $r_{P,\mathrm{DF}}^{★}$ and $r_{C,\mathrm{DF}}^{★}$ are scaled by (2+δ)/(2(1+δ)) subsequently. For DF relay, this maintains the minimum required quality of service for public messages in (24) but leads to modification on the covert rate in (27) as

(35) $$\begin{array}{cccc} \; & r_{P,\mathrm{DF},\mathrm{delay}}^{★}={\bar{r}}_{P},\\ \; & r_{C,\mathrm{DF},\mathrm{delay}}^{★}=\frac{2+\delta }{2\left(1+\delta \right)}\min( & & \log_{2}\left(1+\gamma_{SR,\min}\right)-\frac{2\left(1+\delta \right)}{2+\delta }{\bar{r}}_{P}, \end{array}$$

(36) $$\begin{array}{cccc} \; & \; & & \log_{2}\left(1+\gamma_{RD}\right)-\frac{2\left(1+\delta \right)}{2+\delta }{\bar{r}}_{P},\log_{2}\left(1+\gamma_{RD}\left(1-\bar{\alpha }\right)\right)), \end{array}$$

respectively.

Let us investigate the asymptotic behaviors of the covert rates with the DF relay in (36) and the AF relay in (32).

### 5.1. High Source Transmit Power

For high $P_{S}\to \infty$,

$$\begin{array}{cc} r_{C,\mathrm{DF},\mathrm{delay}}^{★} & ≃\frac{2+\delta }{2\left(1+\delta \right)}\min\left(\log_{2}\left(1+\gamma_{RD}\right)-\frac{2\left(1+\delta \right)}{2+\delta }{\bar{r}}_{P},\log_{2}\left(1+\gamma_{RD}\left(1-\bar{\alpha }\right)\right)\right) \end{array}$$

(37) $$\begin{array}{cc} \; & ≃\frac{2+\delta }{2\left(1+\delta \right)}\log_{2}\left(1+\gamma_{RD}\left(1-\bar{\alpha }\right)\right)\\ r_{C,\mathrm{AF}}^{★} & ≃\min\left(\log_{2}\left(1+\gamma_{RD}\right)-{\bar{r}}_{P},\log_{2}\left(1+\gamma_{RD}\left(1-\bar{\alpha }\right)\right)\right) \end{array}$$

(38) $$\begin{array}{cc} \; & ≃\log_{2}\left(1+\gamma_{RD}\left(1-\bar{\alpha }\right)\right), \end{array}$$

where we used the fact that $\bar{\alpha }\to 1$ for high $P_{S}$ from (23). Comparing (37) and (38), we can thus expect that $r_{C,\mathrm{DF},\mathrm{delay}}^{★}<r_{C,\mathrm{AF}}^{★}$ for high $P_{S}$.

### 5.2. High Relay Transmit Power

For high $P_{R}\to \infty$,

(39) $$\begin{array}{cc} \; & r_{C,\mathrm{DF},\mathrm{delay}}^{★}≃\frac{2+\delta }{2\left(1+\delta \right)}\log_{2}\left(1+\gamma_{SR,\min}\right)-{\bar{r}}_{P}, \end{array}$$

(40) $$\begin{array}{cc} \; & r_{C,\mathrm{AF}}^{★}≃\min\left(\log_{2}\left(1+\gamma_{SR,\min}\right)-{\bar{r}}_{P},\log_{2}\left(1+\gamma_{SR,\min}\left(1-\bar{\alpha }\right)\right)\right). \end{array}$$

We further examine the asymptotic performance for different limits of ${\bar{r}}_{P}$. First, for low ${\bar{r}}_{P}\to 0$,

(41) $$\begin{array}{cc} \; & r_{C,\mathrm{DF},\mathrm{delay}}^{★}≃\frac{2+\delta }{2\left(1+\delta \right)}\log_{2}\left(1+\gamma_{SR,\min}\right), \end{array}$$

(42) $$\begin{array}{cc} \; & r_{C,\mathrm{AF}}^{★}≃\log_{2}\left(1+\gamma_{SR,\min}\left(1-\bar{\alpha }\right)\right). \end{array}$$

We are able to infer that

(43) $$\begin{array}{cc} \; & \frac{2+\delta }{2\left(1+\delta \right)}\log_{2}\left(1+\gamma_{SR,\min}\right)\overset{DF}{\underset{AF}{≷}}\log_{2}\left(1+\gamma_{SR,\min}\left(1-\bar{\alpha }\right)\right)\;\Rightarrow \;\frac{2+\delta }{2\left(1+\delta \right)}\overset{DF}{\underset{AF}{≷}}0, \end{array}$$

since $\bar{\alpha }\to 1$ for typically high DEP threshold ε. “DF” above the inequalities in (43) indicates that $r_{C,\mathrm{DF},\mathrm{delay}}^{★}>r_{C,\mathrm{AF}}^{★}$, and “AF” under them means that $r_{C,\mathrm{DF},\mathrm{delay}}^{★}<r_{C,\mathrm{AF}}^{★}$. Due to δ≥0, we can anticipate that $r_{C,\mathrm{DF},\mathrm{delay}}^{★}>r_{C,\mathrm{AF}}^{★}$.

On the other hand, for high ${\bar{r}}_{P}$,

(44) $$\begin{array}{cc} \; & r_{C,\mathrm{DF},\mathrm{delay}}^{★}≃\frac{2+\delta }{2\left(1+\delta \right)}\log_{2}\left(1+\gamma_{SR,\min}\right)-{\bar{r}}_{P}, \end{array}$$

(45) $$\begin{array}{cc} \; & r_{C,\mathrm{AF}}^{★}≃\log_{2}\left(1+\gamma_{SR,\min}\right)-{\bar{r}}_{P}, \end{array}$$

from which we can draw

(46) $$\begin{array}{cc} \; & \frac{2+\delta }{2\left(1+\delta \right)}\log_{2}\left(1+\gamma_{SR,\min}\right)-{\bar{r}}_{P}\overset{DF}{\underset{AF}{≷}}\log_{2}\left(1+\gamma_{SR,\min}\right)-{\bar{r}}_{P}\;\Rightarrow \;\frac{2+\delta }{2\left(1+\delta \right)}\overset{DF}{\underset{AF}{≷}}1. \end{array}$$

Since δ≥0, it is predicted that $r_{C,\mathrm{DF},\mathrm{delay}}^{★}<r_{C,\mathrm{AF}}^{★}$.

We now proceed with our discussion on high relay transmit power by studying the impact of other parameters.

#### 5.2.1. High Relay Transmit Power with Low Processing Delay

With low δ→0, (39) and (40) reduce to

(47) $$\begin{array}{cc} \; & r_{C,\mathrm{DF},\mathrm{delay}}^{★}≃\log_{2}\left(1+\gamma_{SR,\min}\right)-{\bar{r}}_{P}, \end{array}$$

(48) $$\begin{array}{cc} \; & r_{C,\mathrm{AF}}^{★}≃\min\left(\log_{2}\left(1+\gamma_{SR,\min}\right)-{\bar{r}}_{P},\log_{2}\left(1+\gamma_{SR,\min}\left(1-\bar{\alpha }\right)\right)\right). \end{array}$$

We have two different cases according to the relative value of ${\bar{r}}_{P}$. When ${\bar{r}}_{P}$ is set low, (47) and (48) can be approximated as

(49) $$\begin{array}{cc} \; & r_{C,\mathrm{DF},\mathrm{delay}}^{★}≃\log_{2}\left(1+\gamma_{SR,\min}\right), \end{array}$$

(50) $$\begin{array}{cc} \; & r_{C,\mathrm{AF}}^{★}≃\log_{2}\left(1+\gamma_{SR,\min}\left(1-\bar{\alpha }\right)\right), \end{array}$$

and we predict that $r_{C,\mathrm{DF},\mathrm{delay}}^{★}>r_{C,\mathrm{AF}}^{★}$. In contrast, if ${\bar{r}}_{P}$ is set high, we have

(51) $$\begin{array}{cc} \; & r_{C,\mathrm{DF},\mathrm{delay}}^{★}≃\log_{2}\left(1+\gamma_{SR,\min}\right), \end{array}$$

(52) $$\begin{array}{cc} \; & r_{C,\mathrm{AF}}^{★}≃\log_{2}\left(1+\gamma_{SR,\min}\right). \end{array}$$

It is anticipated that $r_{C,\mathrm{DF},\mathrm{delay}}^{★}=r_{C,\mathrm{AF}}^{★}$ in this case.

#### 5.2.2. High Relay Transmit Power with High Processing Delay

With high δ→∞,

(53) $$\begin{array}{cc} \; & r_{C,\mathrm{DF},\mathrm{delay}}^{★}≃\frac{1}{2}\log_{2}\left(1+\gamma_{SR,\min}\right)-{\bar{r}}_{P}, \end{array}$$

(54) $$\begin{array}{cc} \; & r_{C,\mathrm{AF}}^{★}≃\min\left(\log_{2}\left(1+\gamma_{SR,\min}\right)-{\bar{r}}_{P},\log_{2}\left(1+\gamma_{SR,\min}\left(1-\bar{\alpha }\right)\right)\right). \end{array}$$

We once again encounter different cases depending on ${\bar{r}}_{P}$. When ${\bar{r}}_{P}$ is low, (53) and (54) become close to

(55) $$\begin{array}{cc} \; & r_{C,\mathrm{DF},\mathrm{delay}}^{★}≃\frac{1}{2}\log_{2}\left(1+\gamma_{SR,\min}\right), \end{array}$$

(56) $$\begin{array}{cc} \; & r_{C,\mathrm{AF}}^{★}≃\log_{2}\left(1+\gamma_{SR,\min}\left(1-\bar{\alpha }\right)\right). \end{array}$$

For a typically high DEP threshold that results in $\bar{\alpha }\to 1$. We can thus expect that $r_{C,\mathrm{DF},\mathrm{delay}}^{★}>r_{C,\mathrm{AF}}^{★}$. Next, if ${\bar{r}}_{P}$ is set high, we have

(57) $$\begin{array}{cc} \; & r_{C,\mathrm{DF},\mathrm{delay}}^{★}≃\frac{1}{2}\log_{2}\left(1+\gamma_{SR,\min}\right)-{\bar{r}}_{P}, \end{array}$$

(58) $$\begin{array}{cc} \; & r_{C,\mathrm{AF}}^{★}≃\log_{2}\left(1+\gamma_{SR,\min}\right)-{\bar{r}}_{P}. \end{array}$$

In this case, it is anticipated that $r_{C,\mathrm{DF},\mathrm{delay}}^{★}<r_{C,\mathrm{AF}}^{★}$.

#### 5.2.3. High Relay Transmit Power with Low DEP Threshold

For low ε→0, (23) shows that

(59) $$\begin{array}{c} \bar{\alpha }≃{\left(\max\left(1-\left(\zeta -\frac{1}{\zeta }\right)\frac{{\bar{\sigma }}_{R}^{2}}{2{\left|h_{SR}\right|}^{2}P_{S}},0\right)\right)}^{2}. \end{array}$$

Then, based on the noise uncertainty bound ζ, we draw two different results. First, for low ζ→0dB or 1, we have $\bar{\alpha }\to 1$, and (39) and (40) are approximated by

(60) $$\begin{array}{cc} \; & r_{C,\mathrm{DF},\mathrm{delay}}^{★}≃\frac{2+\delta }{2\left(1+\delta \right)}\log_{2}\left(1+\gamma_{SR,\min}\right)-{\bar{r}}_{P}, \end{array}$$

(61) $$\begin{array}{cc} \; & r_{C,\mathrm{AF}}^{★}≃\log_{2}\left(1+\gamma_{SR,\min}\left(1-\bar{\alpha }\right)\right), \end{array}$$

and $r_{C,\mathrm{DF},\mathrm{delay}}^{★}>r_{C,\mathrm{AF}}^{★}$ is predicted.

On the other hand, with high ζ→∞, we have $\bar{\alpha }\to 0$, and, as a result,

(62) $$\begin{array}{cc} \; & r_{C,\mathrm{DF},\mathrm{delay}}^{★}≃\frac{2+\delta }{2\left(1+\delta \right)}\log_{2}\left(1+\gamma_{SR,\min}\right)-{\bar{r}}_{P}, \end{array}$$

(63) $$\begin{array}{cc} \; & r_{C,\mathrm{AF}}^{★}≃\log_{2}\left(1+\gamma_{SR,\min}\right)-{\bar{r}}_{P}, \end{array}$$

and we can anticipate that $r_{C,\mathrm{DF},\mathrm{delay}}^{★}<r_{C,\mathrm{AF}}^{★}$.

#### 5.2.4. High Relay Transmit Power with High DEP Threshold

For high ε→0.5, (23) reveals that $\bar{\alpha }≃1$. Then,

(64) $$\begin{array}{cc} \; & r_{C,\mathrm{DF},\mathrm{delay}}^{★}≃\frac{2+\delta }{2\left(1+\delta \right)}\log_{2}\left(1+\gamma_{SR,\min}\right)-{\bar{r}}_{P}, \end{array}$$

(65) $$\begin{array}{cc} \; & r_{C,\mathrm{AF}}^{★}≃\log_{2}\left(1+\gamma_{SR,\min}\left(1-\bar{\alpha }\right)\right). \end{array}$$

It is clear that $r_{C,\mathrm{DF},\mathrm{delay}}^{★}>r_{C,\mathrm{AF}}^{★}$ for this case.

### 5.3. Low Relay Transmit Power

For high $P_{R}\to 0$,

(66) $$\begin{array}{cc} \; & r_{C,\mathrm{DF},\mathrm{delay}}^{★}≃\frac{2+\delta }{2\left(1+\delta \right)}\min\left(\log_{2}\left(1+\gamma_{RD}\right)-\frac{2\left(1+\delta \right)}{2+\delta }{\bar{r}}_{P},\log_{2}\left(1+\gamma_{RD}\left(1-\bar{\alpha }\right)\right)\right), \end{array}$$

(67) $$\begin{array}{cc} \; & r_{C,\mathrm{AF}}^{★}≃\min\left(\log_{2}\left(1+\frac{\gamma_{SR,\min}}{\left(\gamma_{SR,\min}+1\right)\gamma_{RD}^{-1}}\right)-{\bar{r}}_{P},\log_{2}\left(1+\frac{\gamma_{SR,\min}\left(1-\bar{\alpha }\right)}{\left(\gamma_{SR,\min}+1\right)\gamma_{RD}^{-1}}\right)\right). \end{array}$$

In addition, for moderate to high $P_{S}$ with $\gamma_{SR,\min}\gg 1$, and (67) is approximately expressed as

(68) $$\begin{array}{cc} \; & r_{C,\mathrm{AF}}^{★}≃\min\left(\log_{2}\left(1+\gamma_{RD}\right)-{\bar{r}}_{P},\log_{2}\left(1+\gamma_{RD}\left(1-\bar{\alpha }\right)\right)\right). \end{array}$$

Thus, $r_{C,\mathrm{DF},\mathrm{delay}}^{★}<r_{C,\mathrm{AF}}^{★}$ is expected.

## 6. Numerical Results

**Figure 2:**
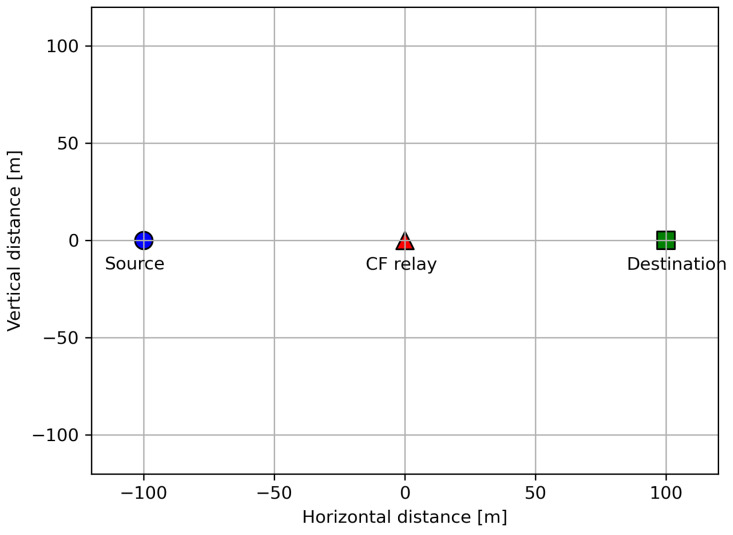
Node placements.

**Figure 3:**
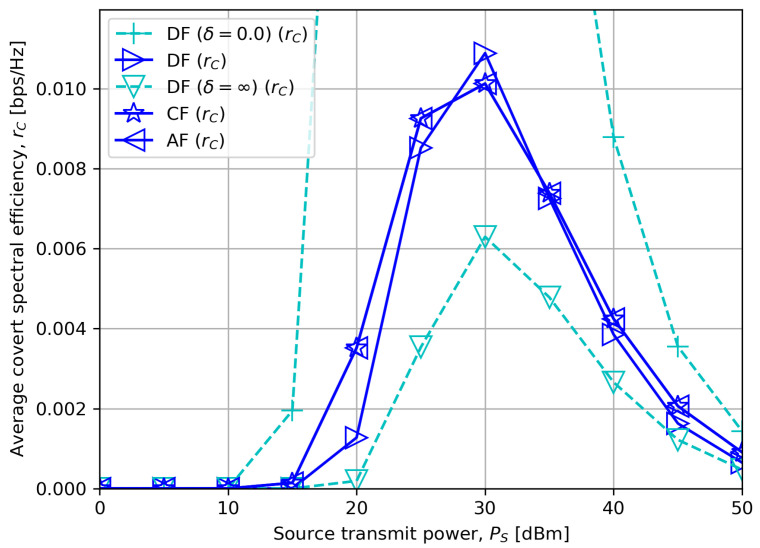
Average covert rate versus source transmit power with δ=5.0.

**Figure 4:**
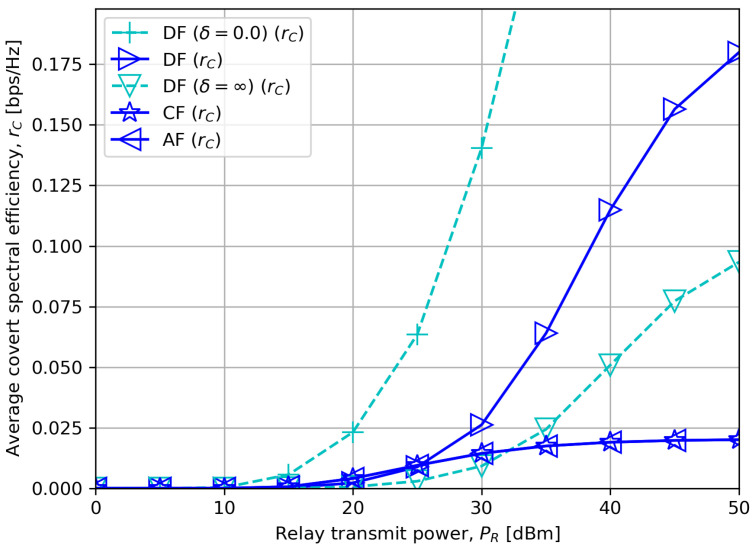
Average covert rate versus relay transmit power with δ=5.0.

**Figure 5:**
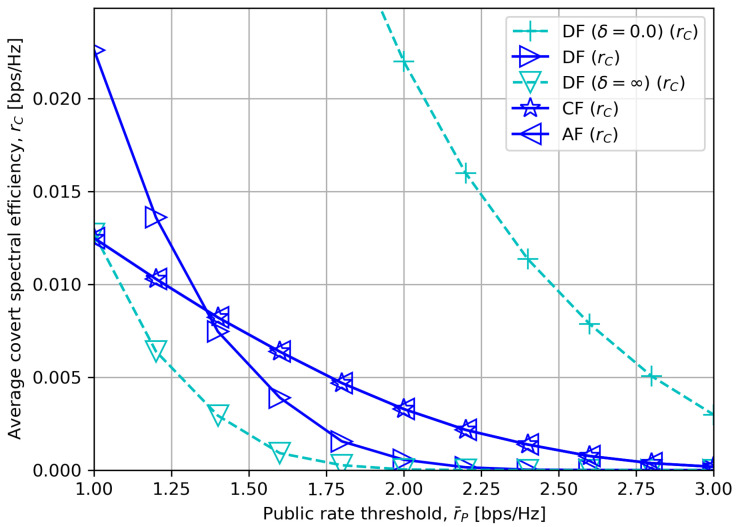
Average covert rate versus minimum quality of service for public messages with δ=5.0.

**Figure 6:**
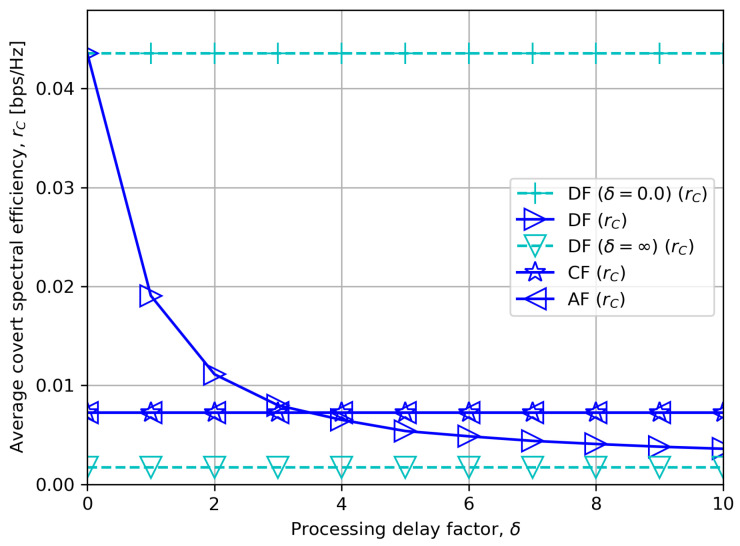
Average covert rate versus processing delay factor.

**Figure 7:**
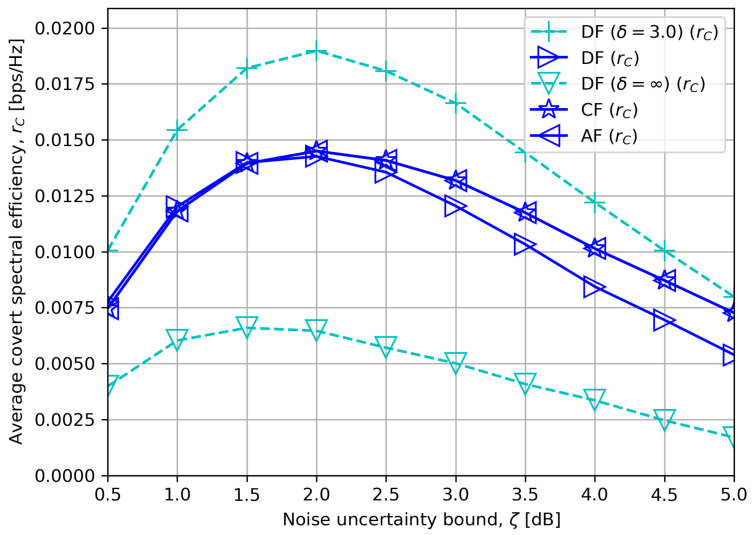
Average covert rate versus noise uncertainty bound with δ=5.0.

**Figure 8:**
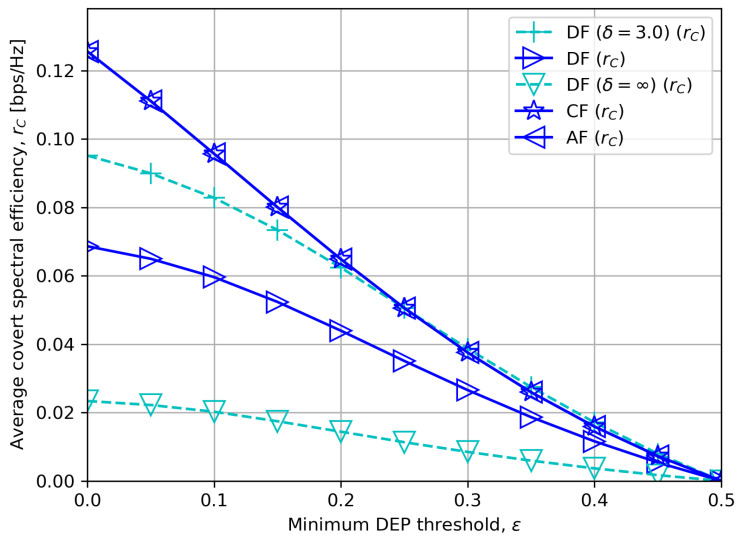
Average covert rate versus minimum DEP threshold with δ=5.0.

**Figure 9:**
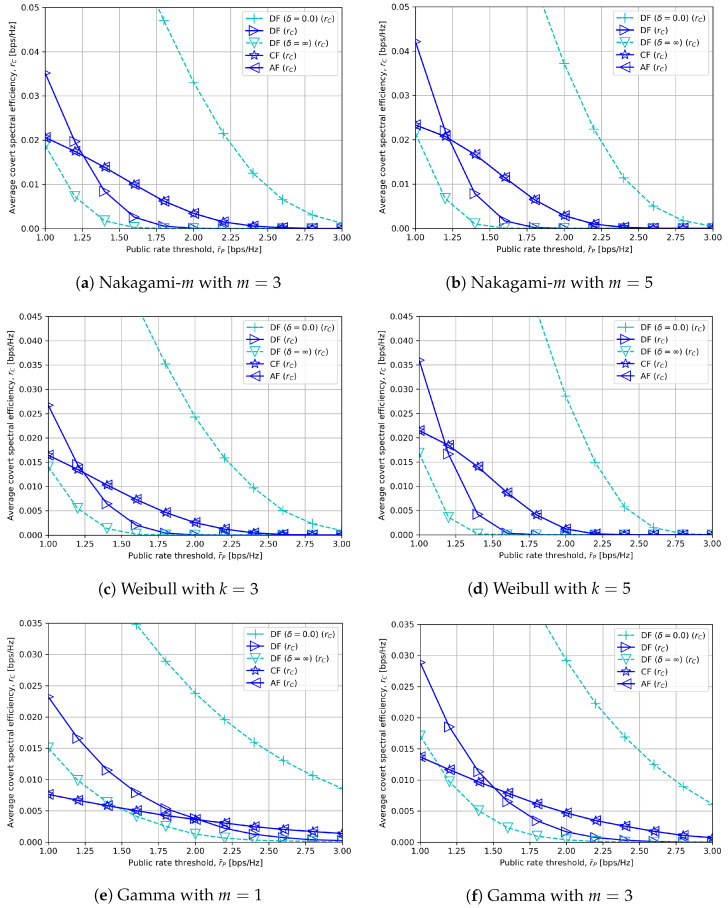
Average covert rate versus minimum quality of service for public messages with δ=5.0 and different channel models.

We assess and compare the covert communication performance in the considered relay systems through numerical simulations. The nodes are placed in a straight line as shown in Figure 2, and the channel coefficient $h_{\mathrm{XY}}$ between node X and Y for X,Y∈{S,R,D} is set to be a function of X-Y distance $d_{\mathrm{XY}}$. [32]. Concretely, we let $h_{\mathrm{XY}}=\sqrt{L_{\mathrm{XY}}}{\hat{h}}_{\mathrm{XY}}$, where $L_{\mathrm{XY}}≜L_{0}{\left(d_{\mathrm{XY}}/d_{0}\right)}^{-\beta }$ means the path loss and the small-scale channel variable ${\hat{h}}_{\mathrm{XY}}$ follows CN(0,1) from Figure 3, Figure 4, Figure 5, Figure 6, Figure 7 and Figure 8 and other distributions in Figure 9, which are be described in detail. $L_{0}$ specifies the path loss at a reference distance $d_{0}=1$ m, β denotes the path loss exponent.

We consider the following system set-ups unless otherwise stated: the bandwidth W=20 MHz, *R*-*S* distance $d_{RS}=100$ m, *R*-*D* distance $d_{RD}=100$ m, source transmit power $P_{S}=23$ dBm, CF relay transmit power $P_{R}=23$ dBm, mean noise power at the CF relay ${\bar{\sigma }}_{R}^{2}=-160$ dBm/Hz, noise uncertainty bound ζ=5 dB, noise power at the destination node $\sigma_{D}^{2}=-160$ dBm/Hz, minimum DEP threshold ε=0.45, pathloss exponent β=3.5, quality of service for public message ${\bar{r}}_{P,D}=1.5$ bps/Hz, and processing delay factor δ=5.0.

Figure 3 shows the average covert rate $r_{C}$ for a different source transmit power $P_{S}$. We first observe that the covert rate of every scheme first increases until a certain $P_{S}$ value and then decreases afterwards. We note that α is lower bounded by $\bar{\alpha }$, and it is preferred to have as low α as possible for a high covert rate. When $P_{S}$ is low, (23) shows that $\bar{\alpha }$ becomes small. Since $r_{C}$ in (6), (9) and (12) are proportional to $P_{S}$, a steady increase in $P_{S}$ has a favorable effect on the improvement of covert rates without excessively increasing $\bar{\alpha }$.

On the other hand, if $P_{S}$ increases beyond a certain value, $\bar{\alpha }$ increases as much as power allocated to the covert message becomes small. This happens since the uncertainty of the noise $\sigma_{R}^{2}$ at the receiver becomes relatively negligible when $P_{S}$ is large, i.e., $\sigma_{R}^{2}\to 0$ in (14). Therefore, even a minimal variation in $P_{S}$ makes the detector decide on the existence of covert transmission.

We can also verify that as $P_{S}$ increases, CF and AF outperform DF, as discussed in Section 5.1. Moreover, as the DF processing delay compared to CF or AF decreases, leading to small δ, the figure shows that DF gradually outperforms the others, which corresponds to the conclusion made in Section 5.2.1.

Figure 4 illustrates the average covert rate in the practical range of $P_{R}$. We notice that DF outperforms CF or AF for high $P_{R}$ since CF or AF compresses or amplifies the noise as well as the actual signal, respectively, which may deteriorate the quality of the received signal at the destination node. In a low $P_{R}$ region, however, CF or AF demonstrates a higher covert rate, which is in accordance with our asymptotic result in Section 5.3.

Figure 5 presents the average covert rate $r_{C}$ for different public rate thresholds ${\bar{r}}_{P}$. It is observed that every relay scheme experiences a decrement in a covert rate since the optimal values in (27), (30) and (32) are negatively related with ${\bar{r}}_{P}$.

We also mark that for a short DF processing delay, DF outperforms CF or AF when ${\bar{r}}_{P}$ is low, but for δ>=5.0, CF or AF exhibits a higher covert rate when ${\bar{r}}_{P}$ is high with moderate $P_{R}=23$ dBm. This corresponds to our analyses in the beginning of Section 5.2 and Section 5.2.2. It is worth noting that, however, the performance of DF, CF and AF tends to converge when ${\bar{r}}_{P}$ is high with a low DF processing delay δ=0, which is discussed in Section 5.2.1.

Figure 6 depicts the average covert rate $r_{C}$ as a function of the processing delay factor δ. The figure shows a straightforward result. Specifically, for an intermediate minimum required quality of service for public messages ${\bar{r}}_{P}=1.5$ bps/Hz, we see that DF outperforms CF or AF for short DF processing delay, and vice versa for a longer DF processing delay.

Figure 7 compares the average covert rate $r_{C}$ in terms of noise uncertainty bound ζ. The covert rates exhibit unimodality. If ζ is low and there is less variance in $\sigma_{R}^{2}$, $\bar{\alpha }$ approaches one, which results in low covertness. A gradual increase in ζ thus improves covertness by confusing the detector. However, a steady increase in ζ beyond a certain ζ begins to have an adverse impact on the covert rates since the worst-case covert rate has an inverse relationship with ζ in (P1), (P2) and (P3). The figure also verifies that DF tends to outperform CF or AF with low DF processing delay, and the opposite is observed for high processing delay.

Figure 8 displays the average covert rate $r_{C}$ for various minimum DEP threshold ε. We see that the covert rates decline if we impose higher DEP requirements. Moreover, with a moderate DF processing delay δ=3.0 and a high noise uncertainty ζ=5 dB, CF or AF outperforms DF when the DEP threshold is low, and vice versa if the DEP threshold is high. This matches our asymptotic analyses in Section 5.2.3 and Section 5.2.4.

Figure 9 provides the average covert rates $r_{C}$ in terms of the public rate threshold ${\bar{r}}_{P}$ under different channel models: Nakagami-*m*, Weibull and gamma fading [33]. Particularly, we may express the small-scale channel gain $|{\hat{h}}_{\mathrm{XY}}|$ in a non-linear form as

(69) $$\begin{array}{c} {\left|{\hat{h}}_{\mathrm{XY}}\right|}^{k}=\sum_{i=1}^{m}\left(H_{1}^{2}+H_{2}^{2}\right), \end{array}$$

where $H_{1}$ and $H_{2}$ are independent and identically distributed Gaussian random variables following $N(0,{\hat{r}}^{k}/\left(2m\right))$ with $\hat{r}≜E[|{\hat{h}}_{\mathrm{XY}}{{|}^{k}]}^{1/k}$ being a *k*-root mean value [34]. It can be shown that $|{\hat{h}}_{\mathrm{XY}}|$ follows Rayleigh fading, as used in the previous figures, by setting k=2 and m=1, Nakagami-*m* fading by k=2, Weibull fading by m=1, and gamma fading by k=1. To ensure the average small-scale channel gain to be normalized by $E[|{\hat{h}}_{\mathrm{XY}}{{|}^{k}]}^{1/k}=1$, we here specify $\hat{r}=1$ for all channel models.

Similar to Figure 5, the covert rates of all relay schemes decline since ${\bar{r}}_{P}$ has a negative effect on the optimal values in (27), (30) and (32) regardless of channel models. Another common observation for δ≥5.0 in Figure 9 is that DF outperforms CF or AF when ${\bar{r}}_{P}$ is low, but CF or AF outperforms DF when ${\bar{r}}_{P}$ is high with moderate $P_{R}=23$ dBm. This also corresponds to the analyses in the beginning of Section 5.2 and Section 5.2.2.

In addition, we can notice that the covert rates are improved for all relay types in the low ${\bar{r}}_{P}$ regime but decline in the high ${\bar{r}}_{P}$ regime when the distribution parameters *m* or *k* increase. The reason for such a phenomenon lies in the fact that *m* or *k* is inversely proportional to the spread in $|{\hat{h}}_{\mathrm{XY}}|$ in each channel model. This means that when *m* or *k* is lower, there is a relatively higher possibility that $|{\hat{h}}_{\mathrm{XY}}|$ is sufficiently strong to support a high ${\bar{r}}_{P}$. In contrast, for a higher *m* or *k*, more stable covert and public rates are achievable with reduced randomness in $|{\hat{h}}_{\mathrm{XY}}|$, but only until a certain ${\bar{r}}_{P}$. To summarize, it becomes easier to meet high ${\bar{r}}_{P}$ when *m* or *k* is lower, while higher and more stable rates can be achieved when *m* or *k* is higher for Nakagami-*m*, Weibull and gamma fading.

## 7. Conclusions

In this paper, we conducted an extensive performance comparison on covert communications among DF, CF and AF relay systems by taking the decoding processing delay into consideration. To this end, we first provided closed-form power distribution solutions between public and covert messages and the corresponding optimal covert rates for each relay protocol, extending our previous results in [24]. Then, we developed delay-aware expressions of the achievable covert rate for each type of relays by adopting the delay relationship in [25]. For high source transmit power or low relay transmit power, our analysis showed that CF or AF tends to outperform DF. For high relay transmit power, various system parameters such as the processing delay, minimum required quality of service for public messages and DEP threshold lead to different performance relationships among DF, CF and AF for high relay transmit power. Numerical results verify our investigation on the performance comparison in various channel models. The results of this paper can provide a useful guideline in an environment where multiple relays with different forwarding protocols exist or where a single relay is capable of selecting either DF, CF or AF. We suggest such covert communications scenarios as interesting future works.

## Data Availability

Not applicable.
